# Effects of weight loss during a very low carbohydrate diet on specific adipose tissue depots and insulin sensitivity in older adults with obesity: a randomized clinical trial

**DOI:** 10.1186/s12986-020-00481-9

**Published:** 2020-08-12

**Authors:** Amy M Goss, Barbara Gower, Taraneh Soleymani, Mariah Stewart, May Pendergrass, Mark Lockhart, Olivia Krantz, Shima Dowla, Nikki Bush, Valene Garr Barry, Kevin R. Fontaine

**Affiliations:** 1 Department of Nutrition Sciences, University of Alabama at Birmingham, 640 Webb Building, 1675 University Blvd, Birmingham, AL 35294-3360 USA; 2 Department of Medicine, Division of Radiology, University of Alabama at Birmingham, Birmingham, AL 35294 USA; 3 Department of Health Behavior, University of Alabama at Birmingham, Birmingham, AL 35294 USA

## Abstract

**Background:**

Insulin resistance and accumulation of visceral adipose tissue (VAT) and intermuscular adipose tissue (IMAT) place aging adults with obesity at high risk of cardio-metabolic disease. A very low carbohydrate diet (VLCD) may be a means of promoting fat loss from the visceral cavity and skeletal muscle, without compromising lean mass, and improve insulin sensitivity in aging adults with obesity.

**Objective:**

To determine if a VLCD promotes a greater loss of fat (total, visceral and intermuscular), preserves lean mass, and improves insulin sensitivity compared to a standard CHO-based/low-fat diet (LFD) in older adults with obesity.

**Design:**

Thirty-four men and women aged 60–75 years with obesity (body mass index [BMI] 30-40 kg/m^2^) were randomized to a diet prescription of either a VLCD (< 10:25:> 65% energy from CHO:protein:fat) or LFD diet (55:25:20) for 8 weeks. Body composition by dual-energy X-ray absorptiometry (DXA), fat distribution by magnetic resonance imaging (MRI), insulin sensitivity by euglycemic hyperinsulinemic clamp, and lipids by a fasting blood draw were assessed at baseline and after the intervention.

**Results:**

Participants lost an average of 9.7 and 2.0% in total fat following the VLCD and LFD, respectively (*p* < 0.01). The VLCD group experienced ~ 3-fold greater loss in VAT compared to the LFD group (− 22.8% vs − 1.0%, *p* < 0.001) and a greater decrease in thigh-IMAT (− 24.4% vs − 1.0%, *p* < 0.01). The VLCD group also had significantly greater thigh skeletal muscle (SM) at 8 weeks following adjustment for change in total fat mass. Finally, the VLCD had greater increases in insulin sensitivity and HDL-C and decreases in fasting insulin and triglycerides compared to the LFD group.

**Conclusions:**

Weight loss resulting from consumption of a diet lower in CHO and higher in fat may be beneficial for older adults with obesity by depleting adipose tissue depots most strongly implicated in poor metabolic and functional outcomes and by improving insulin sensitivity and the lipid profile.

**Trial registration:**

NCT02760641. Registered 03 May 2016 - Retrospectively registered.

## Introduction

Nearly 35% of adults in the U.S. aged 65 and over have obesity, and the prevalence of chronic metabolic disease and impaired functional status among older adults with obesity is particularly high [1, 2]. Expansion of subcutaneous adipose tissue depots may not confer elevated risk per se; rather the accumulation of visceral adipose tissue (VAT), intermuscular adipose tissue (IMAT), and lipid in other ectopic depots may be the most relevant fat depots in determining this risk. VAT and IMAT increase with advancing age, and are linked to insulin resistance and promote a pro-inflammatory state [3–6] that may inhibit skeletal muscle synthesis, contributing to poorer metabolic and functional outcomes [7]. To reduce risk of metabolic disease in older adults with obesity, non-pharmacological interventions designed to deplete visceral and ectopic adiposity and improve insulin resistance are needed.

Typically, individuals with obesity are advised to adopt a calorically restrictive diet to lose fat mass and improve metabolic health. However, some studies suggest that significant weight loss through caloric restriction results in decrease resting metabolic rate and losses of lean tissue, which could possibly lead to accelerated decline in physical function in aging adults [8]. Because the impact of these adverse outcomes on physical function and quality of life warrants further investigation, the recommendation of weight loss to older adults remains controversial [8].

Human and animal studies suggest that change in diet quality may be an appropriate approach to preventing loss of lean tissues while reducing adiposity and improving insulin sensitivity in this population. Diets reduced in carbohydrate (CHO) may be more likely to lower postprandial glucose and insulin secretory response and have been shown to increase hepatic insulin clearance when compared to diets higher in CHO content [9]. Lower insulin secretion and greater clearance may reduce insulin exposure to tissues, ultimately increasing the mobilization of fat from specific adipose tissue depots. We have previously reported significant reductions in VAT and IMAT, as well as maintenance of lean mass, and increased insulin sensitivity during weight maintenance in response to a reduced CHO diet among women with polycystic ovary syndrome (PCOS) [10–12]. These data suggest that a CHO-restricted diet may selectively deplete visceral and ectopic adipose tissue and improve metabolic health, which may be an effective dietary strategy to reduce risk of metabolic disease such as type 2 diabetes (T2D) and cardiovascular (CVD) in older adults with obesity.

Given this, the primary objective of this study was to compare the effects of a very low CHO diet (VLCD) vs. a low-fat diet (LFD) on body composition, fat distribution, and metabolic health in adults aged 60–75 years with obesity.

## Research design and methods

### Participants

Forty men and women with obesity were recruited from the University of Alabama at Birmingham (UAB) EatRight Weight Management Clinic and from the local communities (UAB, Birmingham, and Jefferson and Shelby Counties) via web-based advertisement, flyers, and word-of-mouth from January 2015–July 2017. Inclusion criteria were BMI 30–40 kg/m^2^, age 60–75 years and sedentary (< 2 h/wk. of intentional exercise, and agreed to maintain their level of activity throughout the study). Exclusion criteria included those with diabetes, unwilling to eat the prescribed diets, recent weight change (+/− 10 lbs. in previous year), history of eating disorder, difficulty chewing and swallowing solid food, digestive diseases known to affect nutrient intake, absorption, and metabolism, cognitive impairment, uncontrolled blood pressure (SBP > 159 or DBP > 95 mmHg), history of non-skin cancer in the last 5 years, cardiovascular disease event, severe pulmonary disease; renal failure, major liver dysfunction, current/recent smoker, use of estrogen or testosterone replacement therapy, current use of oral corticosteroids (> 5 d/mth), using medications for treatment of psychosis or manic-depressive illness, and dependence on others for food procurement or preparation. Participants were informed of the experimental design, and oral and written consent was obtained. The study was approved by the Institutional Review Board for Human Use at UAB. The trial is registered on clinicaltrials.gov (NCT02760641).

### Study design

The study design was a randomized two-arm, parallel dietary intervention. Participants were randomized to either 8-weeks of a VLCD (*n* = 20) or a LFD (*n* = 20). All participants underwent medical screening at the UAB EatRight Clinic to confirm eligibility. Dual-energy X-ray absorptiometry (DXA), Magnetic Resonance Imaging (MRI), fasting blood draw, and euglycemic-hyperinsulinemic clamps were performed on all participants at baseline and following completion of the diet intervention. Following completion of baseline testing, participants were assigned to a diet using a block randomization scheme, and the condition assignments were placed in sealed envelopes that were not opened until a specific participant was assigned. Sample size calculations were based on our previous data from a weight maintenance study in a population of men and women with obesity [11]. In this study, we detected a decrease in VAT of 11.0 ± 9.7 cm^2^ after 8 weeks of consumption of a eucaloric reduced-CHO diet. Assuming a change of 11.0 ± 9.7 cm^2^, a two-sided paired t-test, and a significance alpha level of 0.05, we would have over 80% power to detect a significant change in VAT with 17 participants per diet group. Because this was a diet intervention study, it was not possible for participants or all study personnel to be blinded to group assignment; however, study personnel involved in analysis of body composition, fat distribution, and metabolic outcomes were blinded to group assignment.

### Diets

Participants attended weekly individual meetings with a registered dietitian (RD). To provide an incentive to attend regular meetings, and to promote adherence to the diets, participants received breakfast foods compatible with their diet prescription during each visit. VLCD participants received 2 dozen eggs and LFD participants received breakfast bars each week. The number of CHO, protein, and fat servings was determined based on group assignment and total energy requirements as measured by indirect calorimetry (Vmax ENCORE 29 N Systems, SensorMedics Corporation) with an activity factor of 1.35 for women and 1.5 for men. Participants were not instructed to restrict calories, but to reduce either dietary fat or carbohydrate intake based on their randomization. Individualized meal plans were prescribed by the RD to be weight maintaining. The study dietitian would encourage participants to consume enough calories to meet the weight maintaining prescription throughout the intervention. Each diet group received a food list, sample menus, and recipes. During the midpoint of the intervention, 3-day food records (two weekdays and one weekend day) were completed to determine a quantitative measure of adherence to the dietary prescription. A RD analyzed the records using Minnesota Nutrition Data System (NDSR, Software Version 2012).

The VLCD was designed to minimize postprandial glycemic response by providing ≤10% energy from CHO, 25% energy from protein, and ≥ 65% energy from fat. VLCD participants were asked to consume 3 whole eggs (~ 216 kcal, 18.9 g protein, 14.3 g fat, and 1.2 g CHO) per day along with other protein sources including meat, fish, pork, and poultry. The diet emphasized low-glycemic sources of carbohydrate, and included mainly whole foods, such as leafy greens, non-starchy vegetables, some fruits, and high fiber grains with minimal highly processed grain products and added sugar. Fat-containing foods included olive, coconut, and nut oils; butter; tree nuts and nut butters; cheese; cream; coconut milk; and avocados. A number of full-fat dairy products were included. Saturated fat from red meat was `limited to less than 10% of daily caloric intake. Patients obtained the majority of their fat intake from mono-unsaturated fatty acids (e.g. olive oil), and medium-chain triglycerides (e.g., coconut oil and cream); from nuts and nut butters; and from fresh fish.

Participants in the LFD group were counseled to consume the standard, low-fat diet with 55:25:20% energy from CHO:protein:fat. This diet emphasized consuming lean meats, low-fat dairy, whole grains, legumes, fruits and vegetables. The meal plan minimized high-fat foods, high-cholesterol foods, processed starches, and added sugar, and provided < 2300 mg/day sodium. Saturated fat was limited to less than 10% of total energy, and fat-free (or low fat) dairy was recommended. Participants in this group were asked to avoid whole egg consumption during the 8-week intervention period and were provided with breakfast bars to consume as a snack or with a meal each day (~ 180 kcal, 4 g protein, 10 g fat, 22 g CHO). Although this was a high quality, healthful diet, it included a greater amount of carbohydrate foods from such sources as bread, potatoes, and pasta that distinguished it qualitatively from the VLCD. In addition, it had a higher glycemic load than the VLCD.

### Body composition and fat distribution

Total body fat mass and lean mass were measured by DXA (iDXA; GE Healthcare Lunar). Participants were required to wear light clothing, remove all metal objects from their body, and lie supine with arms at their sides while undergoing a total body scan. Subcutaneous abdominal adipose tissue (SAAT), VAT, thigh skeletal muscle (SM), thigh subcutaneous adipose tissue (SAT), thigh perimuscular adipose tissue (PMAT), and thigh-IMAT were determined by magnetic resonance imaging (MRI). Trans-axial abdominal and thigh images were collected using 3D volumetric T1-weighted magnetization-prepared rapid acquisition gradient echo (MPRAGE) using a 1.5-Tesla Philips Achieva system. The echo time, repetition time and pulse flip angles were selected to optimize the signal-intensity contrast between the adipose and non-adipose tissue compartments.

Scans were later analyzed for volume (cm^3^) of adipose tissue and muscle tissue using SliceOmatic image analysis software (version 4.3: Tomovision, Montreal, Canada). The abdomen images from the L1 to the L5 vertebrae were used to analyze VAT and SAAT. Thigh muscle and adipose tissue volume were analyzed using three images from the mid-thigh (mid-point between the anterior iliac crest and the patella). Thigh IMAT, PMAT, and SM were separated from thigh SAT by manually drawing a line along the fascia lata surrounding the thigh muscle. Subsequently, IMAT was partitioned from PMAT and SM by manually drawing a line around the muscle itself to capture adipose tissue located directly between and within muscle groups.

### Hyperinsulinemic-euglycemic clamp

Insulin sensitivity was determined using a hyperinsulinemic euglycemic glucose clamp [13]. Participants were required to fast for 12 h prior to the test. To perform the test, a flexible intravenous catheter was placed in the antecubital space of one arm to infuse insulin and glucose. An additional catheter was placed in the contralateral arm for blood sampling. An insulin solution (regular Humulin, Eli Lilly & Co., Indianapolis, IN) was combined with normal saline and infused at 120 mU/m2min for 4 h using a calibrated syringe pump. Blood glucose concentrations were assessed every 5–10 min using a glucose analyzer and an infusion of 20% dextrose was adjusted to maintain the blood glucose concentration at the fasting level. Blood samples were collected every 10 min for determination of serum insulin concentrations. The 30-min steady state period of the clamp was determined at least 1 h after the beginning of the insulin infusion during which the coefficients of variations for blood glucose and glucose infusion rate were less than 5%. The clamp-derived index of insulin sensitivity was defined as M/(G x ΔI). M is the steady state glucose infusion rate per kg body weight, G is the steady state blood glucose concentrations and Δ1 is the difference between basal and steady state serum insulin concentrations [14].

### Analysis of glucose, hormones and lipids

Analyses were conducted in the Core Laboratory of the Nutrition Obesity Research Center and Diabetes Research Center. Glucose, total cholesterol, HDL-cholesterol and triglycerides were measured using a SIRRUS analyzer (Stanbio Laboratory, Boerne, TX); LDL-C was calculated using the method of Friedewald [15]. The total cholesterol-to-HDL-C ratio was calculated; a ratio of 5 to 1 or lower is the recommended target range, with an optimum ratio of 3·5 to 1. Insulin was assayed by immunofluorescence on a TOSOH AIA-II analyzer (TOSOH Corp., South San Francisco, CA); intra-assay CV of 1·5% and interassay CV of 4·4%.

Circulating markers of inflammation were assessed by immunoassay in fasted morning sera before and after the intervention. High-sensitivity C-reactive protein (CRP) was assessed by turbidometric methods by using a SIRRUS analyzer (Stanbio Laboratory), with reagents obtained from Pointe Scientific, and TNF-α and IL-6 by using electrochemiluminscence (Meso Scale Discovery). Minimum detectable concentrations for each assay were 0.05 mg/L, 0.507 pg/mL, and 0.25 pg/mL, respectively. Mean intra-assay CVs were 7.49, 7.61, and 6.68%, respectively. Mean interassay CVs were 2.13, 5.47, and 9.72%, respectively.

### Resting energy expenditure

REE and respiratory quotient (RQ) were determined at baseline and week 8 after an overnight fast, by indirect calorimetry (Vmax ENCORE 29 N Systems, SensorMedics Corporation, Yorba Linda, CA) in UAB’s Nutrition and Obesity Research Center (NORC) Metabolism Core. A clear, plastic, canopy hood was placed over the head and shoulders, and expired air was collected for 20 min after a 10-min equilibration period. Carbon dioxide production and oxygen consumption were measured continuously during this time.

### Statistical analysis

Descriptive statistics were computed by diet assignment. Statistical tests were two-sided, with an alpha level of 0.05 denoting statistical significance. Analyses were performed using SAS (version 9.1; SAS Institute, Inc., Cary, NC). Paired t-tests were used to determine the difference in baseline and post-intervention body composition, fat distribution, insulin sensitivity, and serum analyte variables by diet group. Analysis of covariance (ANCOVA) determined the effect of diet (adjusted for baseline) on change from baseline to post-intervention in all variables. Further adjustments were made for change in total body fat mass to determine the independent effect of diet on selective depletion of specific adipose tissue depots, changes in insulin sensitivity and other hormones after accounting for weight (fat) loss. Independent t-tests were used to determine group differences in dietary intake.

## Results

Thirty-four women and men completed the study (19 completed the VLCD and 15 completed the LFD). Six participants discontinued the intervention for various reasons unrelated to the study. All 6 participants who dropped out of the study were European American females with ages ranging from 67 to 72. As shown in Table 1, the study participants were ethnically diverse (83 and 17% on the LFD and 92 and 8% on the VLCD were Caucasian and African-American, respectively) with an average age of 71 years in each group. There was no significant difference in BMI, fat mass and lean mass between groups at baseline. Although participants were not instructed to restrict calories, both groups experienced some weight loss. On average, weight loss was significantly greater in the VLCD group compared to the LFD group (− 6.3% vs − 1.0%, *p* < 0.001).

**Table 1:** Baseline characteristics of study participants (Mean ± SD, unless indicated)

Variable	LFD (***n*** = 15)	VLCD (*n* = 19)
Race: (% Caucasian/African-American)	83/17	92/8
Sex: (% Male/Female)	31/69	38/62
Age (yrs)	70.1 ± 2.5	70.3 ± 3.7
Body mass index (kg/m^2^)	34.7 ± 3.8	34.0 ± 3.4
Weight (kg)	96.8 ± 30.9	93.7 ± 15.1
Fat mass (kg)	40.3 ± 7.5	42.4 ± 9.6
Lean mass (kg)	47.3 ± 7.2	50.3 ± 1.2

**Fig. 1:**
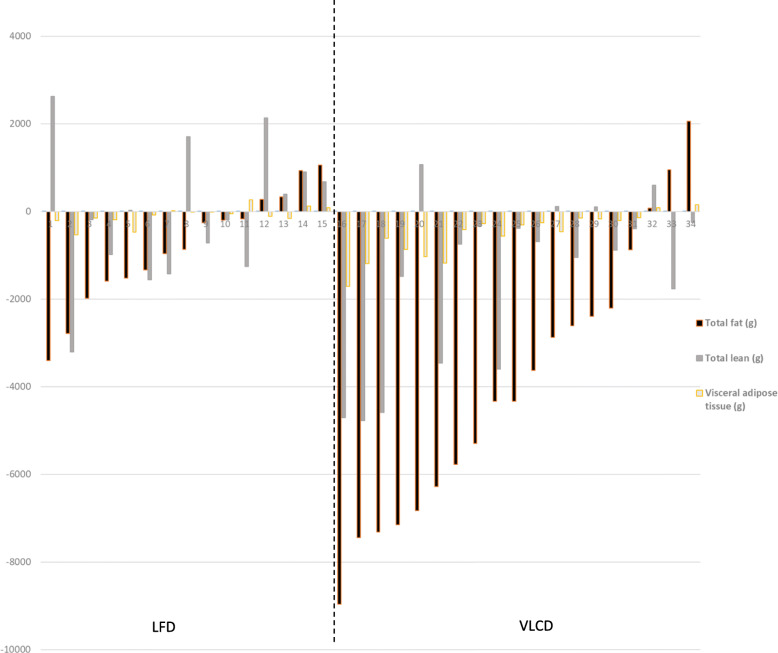
Individual changes in body composition in response to the low fat diet (LFD) and the very low carbohydrate diet (VLCD)

**Fig. 2:**
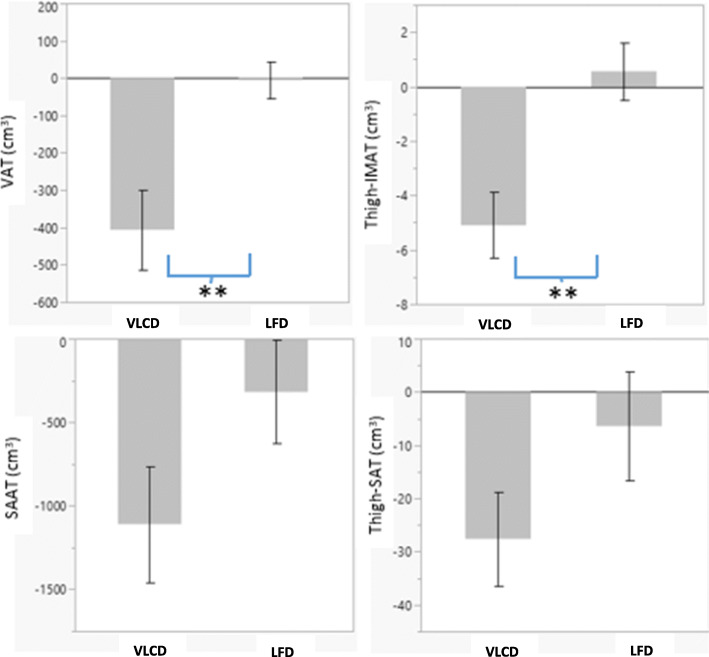
Mean change in adipose tissue depots following consumption of the very low carbohydrate and low fat diets. Participants in the VLCD group experienced 3-fold greater loss of VAT and IMAT when compared to the LFD group. (** indicates *P* < 0.01 for effect of diet)

Changes in body composition and fat distribution by diet group are shown in Table 2. Following the VLCD, there were significant reductions in weight, total fat mass, VAT, SAAT, Thigh-SAT, Thigh-IMAT, and total lean mass. The VLCD group experienced a 9.7% reduction in total fat mass, while the LFD group had a 2.0% reduction (*p <* 0.001). Individual changes in total fat, total lean, and visceral adipose tissue mass are shown in Fig. 1. Changes in VAT, thigh-IMAT, and lean mass were significantly greater in the VLCD vs LFD group (*p* < 0.01). On average, the VLCD decreased VAT by 22.8% vs 1.0% with the LFD and decreased thigh-IMAT by 24.4% vs + 1.0% with the LFD (Fig. 2). There were no significant between group differences in change in Thigh-SM, SAAT, Thigh-SAT and Thigh-PMAT. After adjustment for change in total fat, Thigh-IMAT remained significantly lower in the VLCD group compared to the LFD group and the VLCD group had significantly greater Thigh-SM compared to the LFD group (358.9 ± 5.6 vs 334.4 ± 5.9, respectively).

**Table 2:** Baseline and 8-week body composition and fat distribution outcomes by diet group

	LFD		VLCD		***P*** for diet	***P*** for diet adjusted for change in total fat
	Week 0	Week 8	Week 0	Week 8		
Weight (kg)	96.8 ± 30.9	95.9 ± 28.4	93.7 ± 15.1	87.8 ± 12.5***	< 0.001	0.41
Total fat (kg)	40.3 ± 7.5	39.5 ± 7.5*	42.4 ± 9.6	38.3 ± 10.9***	< 0.001	–
VAT (kg)	1.8 ± 0.8	1.7 ± 0.8	2.2 ± 1.0	1.7 ± 0.7***	< 0.01	.99
VAT (cm^3^)	2077.6 ± 1038	2145.4 ± 1056.8	2613.6 ± 943.5	2249.2 ± 778.2**	0.01	0.58
SAAT (cm^3^)	5496.8 ± 2049	5116.8 ± 2117.3	5481.1 ± 2499.3	4340.9 ± 1626**	0.05	0.15
Thigh SAT (cm^3^)	349.6 ± 225.5	346.2 ± 216.1	317.2 ± 199.7	289.6 ± 186.4**	0.08	0.91
Thigh PMAT (cm^3^)	36.2 ± 16.2	36.4 ± 18.3	33.8 ± 11.3	30.9 ± 13.8	0.13	0.46
Thigh IMAT (cm^3^)	15.1 ± 6.6	15.7 ± 8.7	20.9 ± 13.4	15.8 ± 9.9***	< 0.01	0.02
Thigh SM (cm^3^)	345.9 ± 78.8	339.4 ± 76.3	366.5 ± 95.2	354.3 ± 84.9	0.67	0.02
Total lean (kg)	47.3 ± 7.2	48.0 ± 6.9	50.3 ± 1.2	48.8 ± 1.2**	0.01	0.07

Data presented as mean ± SD

*p < 0.05, **p < 0.01, ***p < 0.001 for paired t-test; *P* for effect of diet, results from ANCOVA (8-week outcome adjusted for baseline); P for the effect of diet, results from ANCOVA (8-week outcome adjusted for baseline and change in fat mass); *VAT* visceral adipose tissue, *SAAT* subcutaneous abdominal adipose tissue, *IMAT* intermuscular adipose tissue, *PMAT* perimuscular adipose tissue, *SAT* subcutaneous adipose tissue, *SM* skeletal muscle

**Fig. 3:**
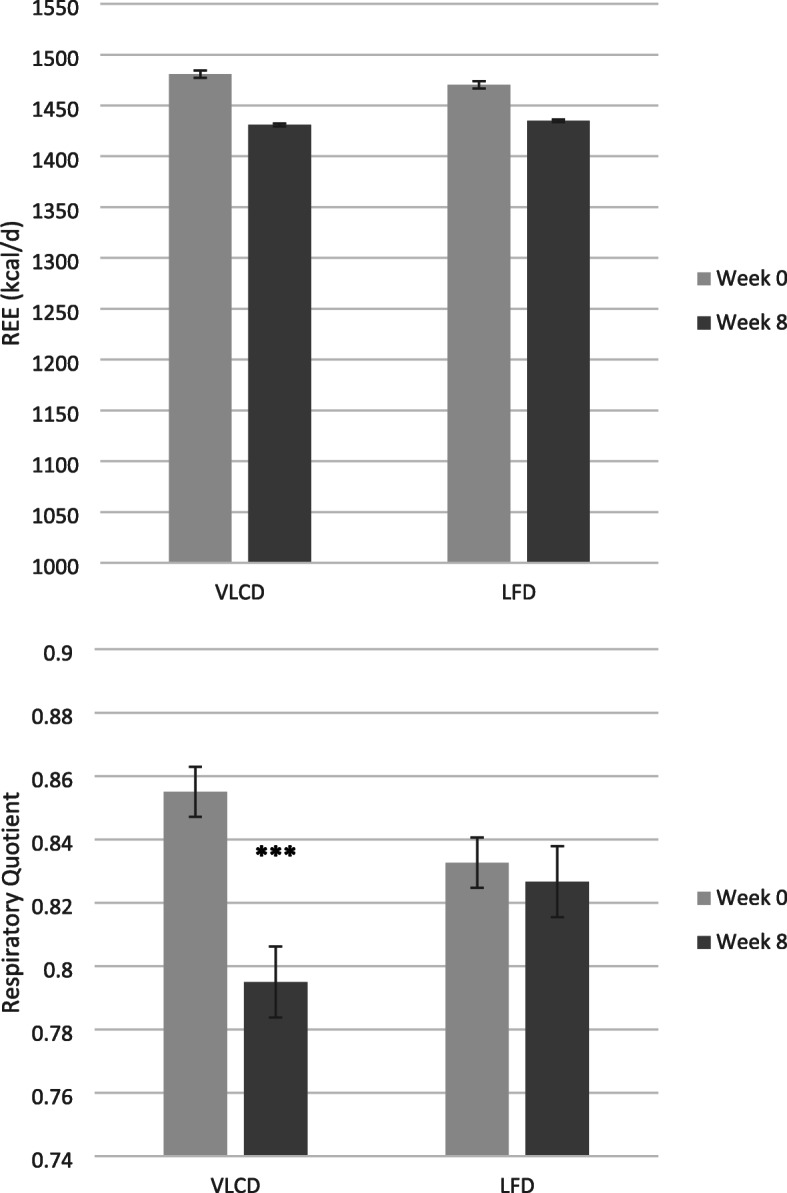
Mean change in resting energy expenditure and respiratory quotient following consumption of the very low carbohydrate and low fat diets. (*** indicates *P* < 0.001)

Changes in metabolic and hormonal outcomes by diet group are shown in Table 3. Following the VLCD, there were significantly greater increases in HDL-C and decreases in HOMA-IR, fasting insulin, and triglycerides compared to the LFD. Within the VLCD group, insulin sensitivity (clamp) significantly increased and there tended to be decreases in total cholesterol and LDL-C. There were no significant changes or between-group differences in change in fasting glucose, hsCRP, TNF-α, IL-6, total cholesterol, and LDL-C. Figure 3 shows the change in REE and RQ from baseline to week 8. There were no significant within-diet group changes in REE or differences between diet groups in change in REE. Among those participants consuming the VLCD, RQ significantly decreased and was significantly lower when compared to those consuming the LFD after 8 weeks (*p* < 0.05 for effect of diet group). Additionally, beta-hydroxybutyrate (BHB) was significantly lower at follow-up compared to the LFD group (0.51 ± 0.39 mM vs 0.13 ± 0.1 mM, *p* < 0.01).

**Table 3:** Baseline and 8-week metabolic outcomes by diet group

	LFD		VLCD		***P*** for diet	***P*** for diet adjusted for change in total fat
	Week 0	Week 8	Week 0	Week 8		
Fasting glucose (mg/dl)	103.4 ± 9.6	101.6 ± 10.6	106.0 ± 13.3	103.4 ± 9.8	0.98	0.65
Fasting insulin (μU/ml)	15.6 ± 6.5	16.0 ± 8.2	13.7 ± 5.6	9.4 ± 4.0*	0.02	0.39
HOMA-IR	4.0 ± 1.6	4.1 ± 2.0	3.4 ± 1.4	2.4 ± 01.1**	0.02	0.42
SI _Clamp_ (10^−4^.kg^−1^.min^−1^/(μU/ml)	1.8 ± 1.0	2.1 ± 1.4	1.7 ± 0.6	2.8 ± 1.7**	0.19	0.26
CRP (mg/l)	3.7 ± 3.7	5.3 ± 5.3	3.2 ± 2.3	5.3 ± 4.9	0.73	0.98
TNF-α (pg/ml)	1.8 ± 0.9	2.0 ± 1.2	1.5 ± 1.0	1.6 ± 0.9	0.71	0.50
IL-6 (pg/ml)	1.6 ± 0.9	1.9 ± 1.1	2.2 ± 1.1	2.1 ± 1.1	0.21	0.13
Cholesterol (mg/dl)	185.5 ± 36.9	183.7 ± 43.0	186.6 ± 40.0	176.0 ± 41.2^†^	0.36	0.83
LDL (mg/dl)	101.9 ± 26.2	103.8 ± 27.4	108.6 ± 34.0	98.4 ± 37.9^†^	0.20	0.94
HDL (mg/dl)	56.0 ± 19.8	56.5 ± 19.4	53.9 ± 10.5	61.2 ± 10.4**	0.04	0.48
Triglycerides (mg/dl)	138.1 ± 47.3	117.2 ± 36.6*	121.1 ± 47.9	81.8 ± 22.9**	< 0.01	0.18

Data presented as mean ± SD

^†^*p* < 0.07, *p < 0.05, **p < 0.01, for paired t-test; *P* for effect of diet, results from ANCOVA (8-week outcome adjusted for baseline); P for the effect of diet, results from ANCOVA (8-week outcome adjusted for baseline and change in fat mass)

Table 4 shows the average participant self-reported dietary intake at the mid-point of the 8 week intervention. There were no significant differences in the grams of total protein and fat consumed per day between the LFD and VLCD diets groups. There was a significant difference in total grams of carbohydrates consumed per day between the two groups. On average, the VLCD group was prescribed 2248 ± 218.1 kkcal /day and the LFD group was prescribed 2137.5 ± 193.7 kkcal/day. The VLCD group reported consuming significantly fewer total calories per day (1114 ± 314 vs 1535 ± 395, *p* < 0.05) than the LFD group. The VLCD group consumed 16:30:54% energy from CHO:protein:fat and the LFD group consumed 47:19:36% energy from CHO:protein:fat.

**Table 4:** Self-reported dietary intake by diet group

	LFD	VLCD	*p*
Total energy (kcal)	1535 ± 395	1114 ± 314	< 0.05
CHO			
% kcal	47 ± 7%	16 ± 7%	< 0.0001
g/day	188 ± 65	48 ± 30	< 0.0001
Protein			
%kcal	18 ± 5%	30 ± 7%	< 0.0001
g/day	69 ± 20	78 ± 15	0.22
Fat			
%kcal	35 ± 7%	54 ± 6%	< 0.0001
g/day	61 ± 18	68 ± 23	0.40
Saturated Fat			
%kcal	13 ± 3%	17 ± 4%	< 0.001
g/day	20 ± 7	22 ± 9	0.57
Alcohol			
%kcal	0.2 ± 0.6%	0.1 ± 0.3%	0.61
g/day	0.4 ± 1.5	0.2 ± 0.6	0.63
Sugar			
Total g/day	78 ± 35	24 ± 21	< 0.001
Added g/day	41 ± 27	13 ± 19	< 0.01
Sodium (mg)	2837.7 ± 702.4	2452.9 ± 709.5	0.16
Glycemic Load	95 ± 33	23 ± 20	< 0.0001

Data reported as Mean ± SD. Results are average intakes from 3-day food records (two-week days and one weekend day) completed after 4 weeks in the study

## Discussion

To our knowledge, this is the first randomized trial comparing the effects of a very low CHO vs standard, low fat diet on change in body composition, fat distribution, and insulin sensitivity using the hyperinsulinemic-euglycemic clamp technique among older adults with obesity. Results indicate that the VLCD group spontaneously restricted intake to a greater extent than the LFD group. As such, when compared to the LFD group, the VLCD group had greater decreases in weight, primarily due to loss of adipose tissue. In 8 weeks, the VLCD group had 3-fold greater decreases in VAT and IMAT compared to LFD group. A direct measure of skeletal muscle via MRI revealed greater thigh-SM among participants consuming the VLCD following adjustment for change in fat mass, suggesting that despite losses of adipose tissue, thigh skeletal muscle was preserved. Even though those consuming the VLCD experienced a significant decrease in lean mass as measured by DXA, there was no difference in change in lean mass between diet groups after adjustment for change in fat mass and hydration status may have influenced the lean mass results [16]. Further, insulin sensitivity was significantly increased among those following 8 weeks of the VLCD. In summary, these data suggest that recommendation of a VLCD in older adults with obesity results in weight loss with favorable changes in body composition, depletion of adipose tissue from metabolically harmful depots, and improvement in lipids and glucose metabolism.

This study provides a unique perspective on the question of whether clinical recommendation of a high quality, CHO- vs fat-restricted diet is effective in improving body composition and fat distribution in older adults with obesity. Each treatment group experienced some benefit (i.e., a modest reduction in total fat mass). However, there were clear benefits in change in body composition in the VLCD group such that there were 5-fold greater reductions in total body fat mass than the LFD group (− 9.7% vs − 2.0%) as a result of greater caloric restriction. Studies examining the effects of intentional weight loss on change in body composition have reported greater loss of fat mass [17, 18] than the present study; however, these studies were of much longer duration (6–12 months). Despite lesser loss of total fat mass, our findings show proportionally greater loss of VAT than these studies suggesting a possible unique effect of a CHO-restricted diet on depletion of these metabolically harmful depots during relatively short-term energy restriction. Rather than total fat mass loss, depletion of VAT likely imparts the greatest cardiometabolic benefit to older adults when considering the well-known associations among this depot, inflammation, and insulin resistance. Thus, the clinical recommendation to consume a CHO-restricted diet to older adults with obesity may be an effective tool to reduce risk of T2D and CVD through the depletion of VAT.

Loss of visceral and ectopic adiposity in response to reduction in CHO intake has been reported in other populations at risk of metabolic disease. We have previously reported significant reductions in VAT among overweight/obese adults in response to a diet reduced in CHO under controlled feeding, weight maintenance conditions [11]. Results indicated that consumption of an 8-week eucaloric reduced CHO/higher fat diet resulted in an 11% loss of VAT when compared to a standard, low-fat diet. In a subsequent crossover study among women with polycystic ovary syndrome (PCOS), similarly we found that consuming a eucaloric reduced CHO/higher fat diet, relative to a standard, low-fat, high CHO diet, resulted in a 5% loss of total fat, a 9% loss of VAT, and an 11% loss of IMAT [10]. Considering that VAT is highly lipolytic, and less susceptible to effects of insulin to suppress lipolysis [19], reducing overall insulin exposure with lower CHO intake may result in the repartitioning of lipid to peripheral, more insulin sensitive depots even in the absence of significant weight loss. In the present study, negative energy balance could explain significant depletion of visceral and ectopic depots; however, it is also possible that decreased carbohydrate intake affected insulin secretion and clearance [9], ultimately permitting greater depletion of ectopic, less insulin sensitive, adipose tissue depots that require greater insulin for maintenance.

This is the first study to report a 24% decrease in IMAT in response to a 8-wk, VLCD intervention in older adults, a population that tends to accumulate high amounts of lipid in the skeletal muscle. These changes would be expected to improve a number of outcomes related to metabolic health and physical function. Multiple longitudinal studies among older adults have reported higher IMAT accumulation to be associated with poorer physical function outcomes over time [6, 20]. Among men with type 2 diabetes (T2D), greater IMAT was associated with greater prevalence of hyperglycemia [21]. Furthermore, greater IMAT has been associated with greater insulin resistance and elevation in markers of inflammation [22]. These observations suggest that fatty infiltration of skeletal muscle may be a contributor to, or a marker of, risk of cardiometabolic disease. Thus, recommendation of a diet reduced in CHO to older adults with obesity may be optimal in order to reduce IMAT and decrease risk of metabolic and physical dysfunction with advancing age.

There were greater losses of lean body mass as measured by DXA in response to the VLCD compared to the LFD. However, measurement of lean body mass via DXA may overestimate change in lean body mass following a VLCD intervention due to changes in hydration status. DXA lean soft tissue includes extra- and intracellular water compartments [16] and it is known that both fasting and a very low carbohydrate diet lead to an accelerated sodium and water excretion through the urine. Very low carbohydrate diets that result in ketosis are also accompanied by glycogen depletion and with it additional water loss. An estimated 400-500 g of glycogen stores can be depleted during a VLCD [23, 24] with water losses of 3-4 g of water for each gram of glycogen [24]. DXA interprets water loss as loss of total lean mass which means following a VLCD, DXA lean mass could decrease by 1.6–2.5 kg due to loss of total body water. In thie present study, we observed an average decrease of − 1.5 kg for DXA total lean mass which may be an overestimation based on published data showing water losses during a weight loss intervention [23]. Thus, DXA lean mass data should be interpreted with caution when hydration status is unknown. Despite the decrease in DXA lean mass in response to the VLCD, a direct measure of thigh skeletal muscle volume via MRI showed no change over 8 weeks. In fact, following adjustment for change in total fat mass, the VLCD group had relatively greater skeletal muscle than the LFD group suggesting preservation of skeletal muscle during weight loss. Preservation of skeletal muscle in response to VLCD may be explained by a reduction in postprandial insulin secretion and an increase in fat oxidation. Very low CHO diets result in elevated ketones produced from metabolism of fatty acids, which act centrally to decrease hepatic glucose production [25]. Due to the decrease in hepatic glucose production, lean mass is spared because fewer amino acids are mobilized from muscle as a substrate for gluconeogenesis. For this reason, VLCDs may be a safe and effective approach to weight loss for older adults with obesity at risk of sarcopenia and functional impairment; however, further studies are needed to explore these possible mechanisms.

The effect of VLCDs on hepatic and peripheral insulin sensitivity in non-diabetic adults is not well understood. Few studies have used the “gold standard” method of measuring insulin sensitivity, i.e. hyperinsulinemic-euglycemic clamp, to measure change in response to the diet. Of those studies, participants had impaired glucose tolerance or T2D, sample sizes were small, and the intervention diet was of short duration [26]. Studies in non-diabetic participants with obesity using surrogate indices of insulin sensitivity have found that the VLCD resulted in significantly lower insulin resistance as measured by HOMA-IR [27–30] and improvement in insulin sensitivity using the QUICKI [31]. In the present study, although it was not powered to detect significant between group differences in SI_Clamp_, there were improvements in insulin sensitivity derived from the clamp in response to the VLCD. Because of the high dose insulin clamp used in this study, it is likely that this measure of insulin sensitivity improved primarily due of an increase in mean peripheral glucose uptake rather than change in hepatic insulin sensitivity. It is possible that the significant reductions in IMAT during the ketogenic diet positively affected skeletal muscle glucose uptake. It has been reported that due to the proximity of IMAT to skeletal muscle tissue, this depot is uniquely positioned to influence muscle insulin sensitivity by exposing the muscle to hormones, adipokines, and excess free fatty acids [32]. Thus, reductions in IMAT in response to the VLCD may confer greater improvements in insulin sensitivity by limiting this exposure. There was also significantly greater improvement in HOMA-IR following the VLCD which is thought to primarily reflect hepatic insulin resistance since this value is derived from fasting glucose and insulin. This data suggest that weight loss in response to a VLCD may improve hepatic insulin resistance to a greater extent than the recommendation of a LFD. Taken together, these data support the use of a VLCD as a means of reducing the risk of metabolic disease related to insulin resistance such as T2D.

In addition to improvements in insulin sensitivity, we observed improvements in the lipid profile in response to the VLCD. Triglycerides (TG) significantly decreased and HCL-C significantly increased to a greater extent following the VLCD vs LFD; however these changes were not independent of change in fat mass. Dramatic decreases in TG and increased HDL-C are common observations in response to low carbohydrate diets and have been reported in numerous randomized clinical trials [33–35]. Even during isocaloric feeding, high carbohydrate diets stimulate de novo lipogenesis (DNL) with proportional increases in plasma TG [35]. The process of hepatic DNL generates fatty acids from other substrates for VLDL synthesis/secretion and indirectly contributes to TG production. Malonyl-CoA is a substrate generated during DNL and inhibits carnitine palmitoyl transferase, the rate limiting enzyme in transporting long chain fatty acids into the mitochondria for oxidation [35]. Conversely, when carbohydrates are restricted, DNL is downregulated and circulating FAs are taken up by the liver and preferentially diverted away TG formation toward mitochondrial oxidation to acetyl CoA [36]. Accumulation of acetyl CoA exceeding the capacity for mitochondrial oxidation results in the formation of ketones [36]. Reduced hepatic production of TG results in less VLDL synthesis and secretion into the circulation. Greater lipolysis of VLDL then results in the formation of HDL-C. Taken together, reduction in circulating TG and increased HDL-C as observed in the present study suggests that consumption of an energy restricted VLCD could be beneficial to the lipid profile in older adults with obesity.

According to the food records, the two interventions resulted in notably different macronutrient intakes and both groups were very close to consuming the recommended %kcal from CHO, protein and fat. During the intervention, the participants in the VLCD group consumed 16% kcal from CHO and more than 54% of their daily calorie intake from fat on average. Yet, because they also restricted energy intake, the amount of total fat (g) they consumed was nearly identical to the LFD diet group. Similarly, there was no significant group difference in total protein intake. Total CHO was the only macronutrient to significantly differ between the two intervention groups, explaining the difference in average calorie intakes. A number of studies have reported similar findings showing that very low CHO diets induce spontaneous energy restriction due to their effects on appetite and feelings of hunger [37, 38]. Additionally, RQ significantly decreased over 8 weeks among those consuming the VLCD indicating a shift in substrate oxidation. A decrease in RQ would be expected with the consumption of an energy restricted, proportionally higher fat diet and supports the self-reported intake data. Additionally, BHB, a ketone synthesized in the liver from fatty acids, was significantly higher in the VLCD group after 8 weeks of the intervention. Elevated BHB provides further objective evidence that participants were consuming a low carbohydrate diet.

There are limitations and strengths to our study. Because the two intervention diets were not isocaloric, we cannot draw conclusions regarding whether the effects of the VLCD were superior to an energy restricted, LFD on change in body composition and fat distribution independent of weight loss among adults with obesity. We did not provide all food for the participants; however, we did provide eggs for the VLCD group and breakfast bars to the LFD group for daily consumption. Provision of all food could have increased dietary adherence, study visit attendance, and/or participant retention. Adherence to the diet prescriptions was assessed using self-reported, 3-day food record at the midpoint of the intervention, which may not be accurate or representative of food intake across the entire intervention; however, adherence was supported by objective measures of macronutrient intake, RQ and BHB. Because of feasibility issues, we did not blind participants or study staff to diet assignment with the resultant potential for bias. However, staff performing DXA and MRI analysis were blinded to diet assignment and the intervention measurements were performed as objectively as possible.

In conclusion, the VLCD induced significant weight loss and reduced VAT and thigh-IMAT in a group of older adults with obesity. Depletion of these adipose tissue depots may be critical for reducing the risk of metabolic disease with advancing age. Furthermore, despite significant loss of fat mass, those consuming the VLCD retained greater thigh skeletal muscle volume. Weight loss in response to a very low CHO, energy restricted diet also improved hepatic and peripheral insulin sensitivity suggesting a reduced risk of cardiometabolic disease.

## Data Availability

The datasets used during the present study are available from the corresponding author upon reasonable request.

## Abbreviations

CHO: Carbohydrate
NIH: National Institutes of Health
BMI: Body mass index
LFD: Low fat diet
VLCD: Very low carbohydrate diet
PCOS: Polycystic ovary syndrome
DXA: Dual energy x-ray absorptiometry
MRI: Magnetic resonance imaging
NDSR: Nutrition Data System for Research
VAT: Visceral adipose tissue
SAAT: Subcutaneous abdominal adipose tissue
IMAT: Intermuscular adipose tissue
SAT: Subcutaneous adipose tissue
PMAT: Perimuscular adipose tissue
CRU: Clinical Research Unit
